# The Survey of H5N1 Flu Virus in Wild Birds in 14 Provinces of China from 2004 to 2007

**DOI:** 10.1371/journal.pone.0006926

**Published:** 2009-09-09

**Authors:** Zheng Kou, Yongdong Li, Zuohua Yin, Shan Guo, Mingli Wang, Xuebin Gao, Peng Li, Lijun Tang, Ping Jiang, Ze Luo, Zhi Xin, Changqing Ding, Yubang He, Zuyi Ren, Peng Cui, Hongfeng Zhao, Zhong Zhang, Shuang Tang, Baoping Yan, Fumin Lei, Tianxian Li

**Affiliations:** 1 State Key Laboratory of Virology, Wuhan Institute of Virology, Chinese Academy of Sciences, Wuhan, China; 2 Key Laboratory of Zoological Systematics and Evolution, Institute of Zoology, Chinese Academy of Sciences, Beijing, China; 3 Institute of Remote Sensing Applications, Chinese Academy of Sciences, Beijing, China; 4 Department of Microbiology, Anhui Medical University, Hefei, China; 5 Shaanxi Institute of Zoology, Xian, China; 6 Yanan Hospital, Kunming, China; 7 Hubei Center for Disease Control and Prevention, Wuhan, China; 8 College of Veterinary Medicine, Nanjing Agricultural University, Nanjing, China; 9 Information Center of Computer Network, Chinese Academy of Sciences, Beijing, China; 10 Qinghai Lake National Nature Reserve, Xining, China; 11 Yuyao Institute of Animal Diseases, Ningbo, China; 12 College of Life Science, Shaanxi Normal University, Xian, China; University of Oxford, United Kingdom

## Abstract

**Background:**

The highly pathogenic H5N1 avian influenza emerged in the year 1996 in Asia, and has spread to Europe and Africa recently. At present, effective monitoring and data analysis of H5N1 are not sufficient in Chinese mainland.

**Methodology/Principal Findings:**

During the period from April of 2004 to August of 2007, we collected 14,472 wild bird samples covering 56 species of 10 orders in 14 provinces of China and monitored the prevalence of flu virus based on RT-PCR specific for H5N1 subtype. The 149 positive samples involved six orders. Anseriformes had the highest prevalence while Passeriformes had the lowest prevalence (2.70% versus 0.36%). Among the 24 positive species, mallard (*Anas platyrhynchos*) had the highest prevalence (4.37%). A difference of prevalence was found among 14 provinces. Qinghai had a higher prevalence than the other 13 provinces combined (3.88% versus 0.43%). The prevalence in three species in Qinghai province (Pintail (*Anas acuta*), Mallard (*Anas platyrhynchos*) and Tufted Duck (*Aythya fuligula*)) were obviously higher than those in other 13 provinces. The results of sequence analysis indicated that the 17 strains isolated from wild birds were distributed in five clades (2.3.1, 2.2, 2.5, 6, and 7), which suggested that genetic diversity existed among H5N1 viruses isolated from wild birds. The five isolates from Qinghai came from one clade (2.2) and had a short evolutionary distance with the isolates obtained from Qinghai in the year 2005.

**Conclusions/Significance:**

We have measured the prevalence of H5N1 virus in 56 species of wild birds in 14 provinces of China. Continuous monitoring in the field should be carried out to know whether H5N1 virus can be maintained by wild birds.

## Introduction

Influenza A virus contains eight segments of single-strand negative RNA. Segment 4 codes hemagglutinin (HA) gene and segment 6 codes neuraminidase (NA) gene. According to the antigenic characteristics of HA and NA, avian influenza A virus has 16 subtypes HA and nine subtypes NA [1]. Water fowl is regarded as the nature reservoir of Influenza A virus [2]–[4].

Highly pathogenic H5N1 bird flu emerged in the year 1996 in Asia [5]. Large-scale culling on domestic fowl has been carried out in order to avoid the further outbreak of bird flu. It was believed before 1997 that avian influenza virus could not infect humans directly until the genes of avian influenza virus mixed with those of human viruses, which was deduced by swine, the intermediate host [2]. However, the fact that H5N1 bird flu virus was isolated from the sample of patients suffering from flu in the year 1997 changed people's opinion. Furthermore, it is proved that H5N1 bird flu virus can infect the respiratory system of human, which can lead to death by crossing over the boundary of species directly [6]–[8].

From 2003, there have been a series of infections of humans by H5N1 bird flu virus in Asia, and the reports of death have aroused the extensive attention among the general public [9]–[12]. Large-scale culling on domestic fowl has been carried out in many countries in order to avoid further outbreak of avian flu. In 2005, a large number of wild birds in Qinghai Lake, China, were infected and killed by highly pathogenic H5N1 virus [13]–[16]. It is estimated that the number of bar-headed goose decreased by 5%–10% across the world, resulting from this epizootic disease alone. Apart from the continuous spread in China and other parts of Asia, this kind of virus also appeared in more than 60 countries across Eurasia and Africa [17]–[20]. According to the time and location of the outbreak of bird flu and the migration time of migratory birds, it is doubted that the spread of bird flu might be related to the migration of migratory birds across the globe [21]–[23].

China is in a critical position along the migration routes for the migratory birds across Eurasia. At present, effective monitoring and data analysis of H5N1 virus among wild birds are quite insufficient in Chinese mainland. During the period from April, 2004 to August, 2007, we collected the samples from wild birds in 14 provinces in Chinese mainland and monitored H5N1 virus based on reverse transcription-polymerase chain reaction (RT-PCR). We also discuss the prevalence of H5N1 virus in wild birds in Chinese mainland.

## Results

### Survey on H5N1 virus in wild birds

**Figure 1:**
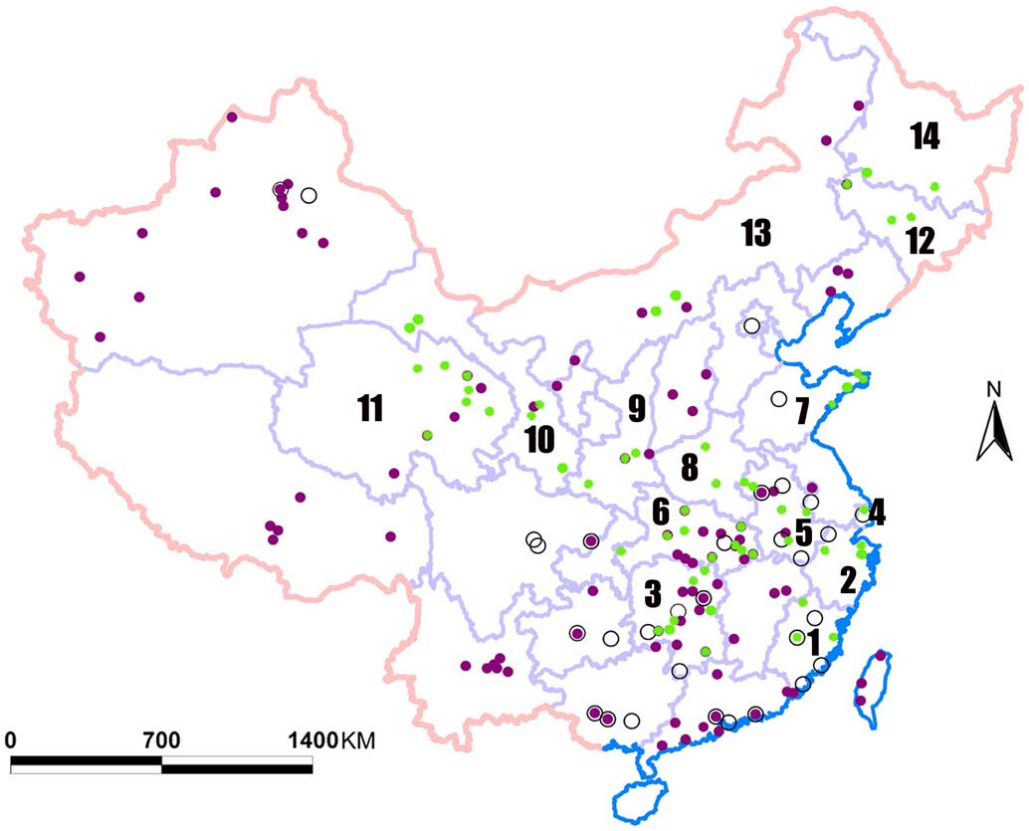
Distribution of collected samples from wild birds in Chinese mainland. The 14 provinces are marked by numbers: 1 = Fujian; 2 = Zhejiang; 3 = Hunan; 4 = Shanghai; 5 = Anhui; 6 = Hubei; 7 = Shandong; 8 = Henan; 9 = Shannxi; 10 = Gansu; 11 = Qinghai; 12 = Jilin; 13 = Neimenggu; 14 = Heilongjiang. Green dots represent locations of samples from wild birds. Purple dots represent locations of H5N1 confirmed cases of poultry or migratory bird (ref 26). Hollowed circles represent locations of H5N1 confirmed cases of Human (ref 27).

We launched a sample collection from the wild birds living in the major lakes and wetlands covering 14 provinces of China from 2004 to 2007 (Figure 1). The samples were cloacal swab, organ tissues or fresh excrement from the gathering place of a bird flock of a single species. We totally collected 14,472 wild bird samples covering 56 species of 10 orders (Table 1). All the samples were immediately put into small tubes containing transferring solution and then stored in liquid nitrogen container within 2 hours. After tested by RT-PCR, 149 samples were confirmed to be positive for H5N1 subtype. By adopting the method based on chick embryo, 17 virus strains have been isolated from positive samples.

**Table 1:** Overview of wild bird samples involved in the study.

Order	Family	Species (n)	Sampled (n)
Anseriformes	Anatidae	10	3,145
Charadriiformes	Scolopacidae	1	746
Columbiformes	Columbidae	1	571
Coraciiformes	Upupidae	1	15
Gruiformes	Gruidae	1	68
Lariformes	Charadriidae	1	95
	Laridae	4	1,842
	Scolopacidae	1	136
Passeriformes	Alaudidae	4	722
	Corvidae	2	448
	Emberizidae	2	489
	Fringillidae	2	565
	Paradoxornithidae	1	325
	Paridae	5	894
	Passeridae	2	1,620
	Pycnonotidae	2	479
	Pyrgilauda	1	25
	Sylviidae	2	215
	Timallidae	2	639
	Turdidae	8	899
Pelecaniformes	Phalacrocoracidae	1	347
Podicipediformes	Podicipedidae	1	158
Strigiformes	Strigidae	1	29
**Total: 10**	**23**	**56**	**14,472**

149 samples were H5/N1 positive after the test based on RT-PCR (Table 2). The positive samples involved 24 species of six orders (nine species from Anseriformes, one specie from Charadriiformes, one specie from Columbiformes, three species from Lariformes and nine species from Passeriformes, one specie from Pelecaniformes). The samples of Anseriformes had the highest prevalence and those of Passeriformes had the lowest prevalence (2.70% versus 0.36%, Pearson χ^2^-test, ρ<0.001).

**Table 2:** Prevalence of H5N1 virus in wild birds in 14 provinces of China.

Order	Common name	Species	Sampled (n)	Positive (n)	Rate (%)
Anseriformes	Ducks	6 species			
		Common Teal (*Anas crecca*)	161	5	3.11
		Mallard (*Anas platyrhynchos*)	458	20	4.37
		Pintail (*Anas acuta*)	487	20	4.11
		Red-crested Pochard (*Rhodonessa rufina*)	68	2	2.94
		Ruddy Shel Duck (*Tadorna ferruginea*)	329	5	1.52
		Tufted Duck (*Aythya fuligula*)	524	9	1.72
	Geese	2 species			
		Bar-headed Goose (*Anser indicus*)	675	13	1.93
		Lesser White-fronted Goose (*Anser erythropus*)	143	3	2.10
	Swans	1 species			
		Whooper Swan (*Cygnus cygnus*)	223	8	3.59
Charadriiformes	Curlews	1 species			
		Eurasian Curlew (*Numenius arquata*)	746	3	0.40
Columbiformes	Pigeons	1 species			
		Rock Dove (*Columba livia*)	571	6	1.05
Lariformes	Gulls	3 species			
		Brown-headed Gull (*Larus brunnicephalus*)	670	16	2.39
		Pallas's Gull (*Larus ichthyaetus*)	507	6	1.18
		Slaty-backed Gull (*Larus schistisagus*)	298	1	0.34
Passeriformes	Bulbul	1 species			
		Madagascar Bulbul (Hypsipetes leucocephalus)	385	2	0.52
	Finchs	1 species			
		Plain Mountain Finch(*Leucosticte nemoricola*)	209	1	0.48
	Fulvettas	1 species			
		Grey-cheeked Fulvetta (*Alcippe morrisonia*)	285	2	0.70
	Larks	1 species			
		Eurasian Sky lark(*Alauda arvensis*)	114	1	0.88
	Thrushs	1 species			
		Scaly Thrush (*Zoothera dauma*)	273	2	0.73
	Tits	1 species			
		Green-backed Tit (*Parus monticolus*)	166	1	0.6
	Sparrows	2 species			
		Rock Petronia (*Petronia petronia*)	244	1	0.41
		Tree Sparrow (*Passer montanus*)	1,376	15	1.09
	Yuhinas	1 species			
		White-collared Yuhina (*Yuhina diademata*)	354	1	0.28
Pelecaniformes	Cormorants	1 species			
		Great Cormorant (Phalacrocorax carbo)	347	6	1.73

### Prevalence of H5N1 virus in Anseriformes

The positive samples of Anseriformes were collected from ducks, geese and swans. The prevalence among 9 species of Anseriformes varied from 1.52% to 4.37% and did not show obvious difference. Ruddy Shel Duck (*Tadorna ferruginea*) had the lowest prevalence (1.52%), while Mallard (*Anas platyrhynchos*) had the highest prevalence (4.37%). Four strains of H5N1 subtype were isolated from Anseriformes, which were named as: A/wDk/Shanghai/59/04 (H5N1), A/BarHeadedGoose/Qinghai/F/06 (H5N1), A/Swan/Qinghai/01/06 (H5N1) and A/BarHeadedGoose/Qinghai/369/07 (H5N1) respectively. In Qinghai province, two strains of virus were isolated from the samples of bar-headed goose from 2006 to 2007 and one strain of virus was isolated from the samples of swan in the year 2006.

### Prevalence of H5N1 virus in Lariformes

The positive samples of Lariformes were only collected from gulls. The prevalence of Brown-headed Gull (*Larus brunnicephalus*) was relatively high (2.39%). Three H5N1 viruses were isolated from Lariformes, which were named as: A/BrownHeadedGull/Qinghai/3/06 (H5N1), A/SlatyBackedGull/Shandong/59/04 (H5N1) and A/PallasGull/Qinghai/81/07 (H5N1). Among the samples of Anseriformes and Lariformes, five H5N1 viruses were isolated from Qinghai provinces from 2006 to 2007, which suggested that wild birds in Qinghai province might maintain H5N1 virus.

### Prevalence of H5N1 virus in Passeriformes

We collected 7,320 samples from the 33 species of Passeriformes (Table 1). The prevalence of Passeriformes was 0.36% and was obviously lower than Anseriformes as mentioned above. The 26 positive samples distributed in 9 species. The prevalence (1.09%) of Tree Sparrow (*Passer montanus*) was relatively high among all. Eight H5N1 viruses were isolated from the 26 positive samples and they were named as: A/TreeSparrow/Henan/1/04 (H5N1), A/TreeSparrow/Henan/2/04 (H5N1), A/TreeSparrow/Henan/3/04 (H5N1), A/TreeSparrow/Henan/4/04 (H5N1), A/Babbler/Fujian/320/04(H5N1), A/BlackBird/Hunan/1/04 (H5N1), A/GoldenMountainThrush/Fujian/376/04 (H5N1), and A/BlackBulbul/Fujian/439/04 (H5N1).

### Prevalence of H5N1 virus in other bird species

Samples of Columbiformes were only collected from Rock Dove (*Columba livia*). Six samples were H5N1 positive and the prevalence is 1.05%, which is similar to that of Tree Sparrow (*Passer montanus*). One H5N1 virus was isolated and named as: A/Pigeon/Zhejiang/17/05(H5N1). The samples of Charadriiformes were only colleted from Eurasian Curlew (Numenius arquata) and the prevalence is 0.40%. One H5N1 virus was isolated (A/Curlew/Shandong/61/04 (H5N1)). The prevalence of Pelecaniformes was 1.73%, but no virus was isolated. It was noted that H5N1 viruses (A/Curlew/Shandong/61/04, A/wDk/Shanghai/59/04 and A/SlatyBackedGull/Shandong/59/04) were isolated from the samples of migratory bird in Shanghai and Shandong province in the year 2004 and the sites for samples are located in coastal wetlands which belong to the Asia-Australia fly way.

### Location of positive samples

A certain difference of prevalence was found among 14 provinces as shown in table 3. Qinghai had a higher prevalence than other 13 provinces combined (3.88% versus 0.43%, Pearson 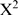-test, ρ<0.001). Qinghai is a crucial intersection in the fly way of migratory birds and a large number of wild birds stay there every year. Most of the samples in Qinghai province were collected from migratory birds, which suggested that the high prevalence might relate to migratory birds. Most of the samples in Henan province were those from Tree Sparrow (*Passer montanus*). The relative high prevalence in Henan might be related with the high prevalence of Tree Sparrow. As shown in figure 1, most H5N1 positive provinces (Qinghai, Gansu, Shanghai, Zhejiang, Neimenggu, Shandong, Shannxi, Fujian, Hunan) had poultry or human H5N1 cases, which suggested that these cases might be related with wild birds.

**Table 3:** Prevalence of H5N1 virus in 14 provinces of China.

Area	Province	Sampled(n)	Positive(n)	Rate(%)
Central	Henan	980	14	1.43%
	Hunan	1531	6	0.39%
	Hubei	1122	0	0.00%
East	Anhui	821	0	0.00%
	Zhejiang	889	3	0.34%
	Fujian	1459	10	0.69%
	Shandong	1131	3	0.27%
	Shanghai	716	2	0.28%
Northwest	Shaanxi	1159	6	0.52%
	Gansu	851	4	0.47%
	Qinghai a	2684	98	3.65%
Northeast	Heilongjiang a	272	0	0.00%
	Jilin a	187	0	0.00%
North	Neimenggu a	670	3	0.45%

a No samples were collected in 2004.

### Screened results of Qinghai samples

Qinghai province, located on the Qinghai-Tibet plateau of Northwest China, had special environments and various wildlife species. According to pervious study [3], three mainly international flyways intercross at Qinghai province: the Central Asia flyway, the East Asia-Australia flyway and East Africa-West Asia flyway. In April, 2005, highly pathogenic avian influenza H5N1 virus broke out in wild bird populations in Qinghai Lake [13], [14], a major breeding site for migratory birds. It was the first report that highly pathogenic avian influenza H5N1 viruses were isolated from the migratory birds. From Sep 2005 to Sep 2007, we collected 2,684 samples from Qinghai province. 95 of 98 positive samples were collected from Anseriformes, Lariformes and Pelecaniformes.

As shown in table 4, the prevalence of 11 species of Anseriformes, Lariformes and Pelecaniformes varied from 2.17% to 11.19%. Mallard (*Anas platyrhynchos*) had the highest prevalence (11.19%) while Ruddy Shelduck (*Tadorna ferruginea*) had the lowest prevalence (2.17%). The prevalence of three species in Qinghai province (Pintail (*Anas acuta*), Mallard (*Anas platyrhynchos*) and Tufted Duck (*Aythya fuligula*)) were obviously higher than those in other 13 provinces (Pearson χ^2^-test, ρ<0.001). Except Common Teal (*Anas crecca*) and Red-crested Pochard (*Rhodonessa rufina*), the prevalence of other six species were relatively higher than those in other 13 provinces.

**Table 4:** Prevalence of H5N1 virus in wild birds of Anseriformes, Lariformes, Pelecaniformes in Qinghai Province.

Order	Common name	Species	Rate only for Qinghai (%)	Rate for other 13 provinces (%)
Anseriformes	Ducks	6 species		
		Pintail (Anas acuta)	9.84 (18/183)	0.66(2/304)
		Common Teal (Anas crecca)	3.11 (5/161)	-a
		Ruddy Shelduck (Tadorna ferruginea)	2.17 (3/138)	1.05(2/191)
		Mallard (Anas platyrhynchos)	11.19 (15/134)	1.54(5/324)
		Tufted Duck (Aythya fuligula)	7.09 (9/127)	0.00(0/397)
		Red-crested Pochard (Rhodonessa rufina)	2.94 (2/68)	-a
	Geese	1 species		
		Bar-head Goose (Anser indicus)	2.27 (12/528)	0.68(1/147)
	Swans	1 species		
		Whooper Swan (Cygnus cygnus)	4.00 (6/150)	2.74(2/73)
Lariformes	Gulls	2 species		
		Brown-headed Gull (Larus brunnicephalus)	3.60 (14/389)	0.71(2/281)
		Pallas's Gull (Larus ichthyaetus)	2.34 (5/214)	0.34(1/293)
Pelecaniformes	Cormorants	1 species		
		Great Cormorant (Phalacrocorax carbo)	3.92 (6/153)	0.00(0/194)

a No samples were collected in other 13 provinces.

### Sequence analysis of HA genes

17 strains of H5N1 virus were isolated from 149 positive samples covering 24 species. We sequenced the HA genes of these 17 strains. Based on the results of sequence analysis, it was known that all of the HA proteins had a series of basic amino acids at the HA cleavage site (RRRKKR/G), which is a characteristic of highly pathogenic influenza viruses. All amino acids relevant to receptor binding (amino acids 99, 130–134, 149, 151, 186, 190–191, and 220–225) did not have mutations.

**Figure 2:**
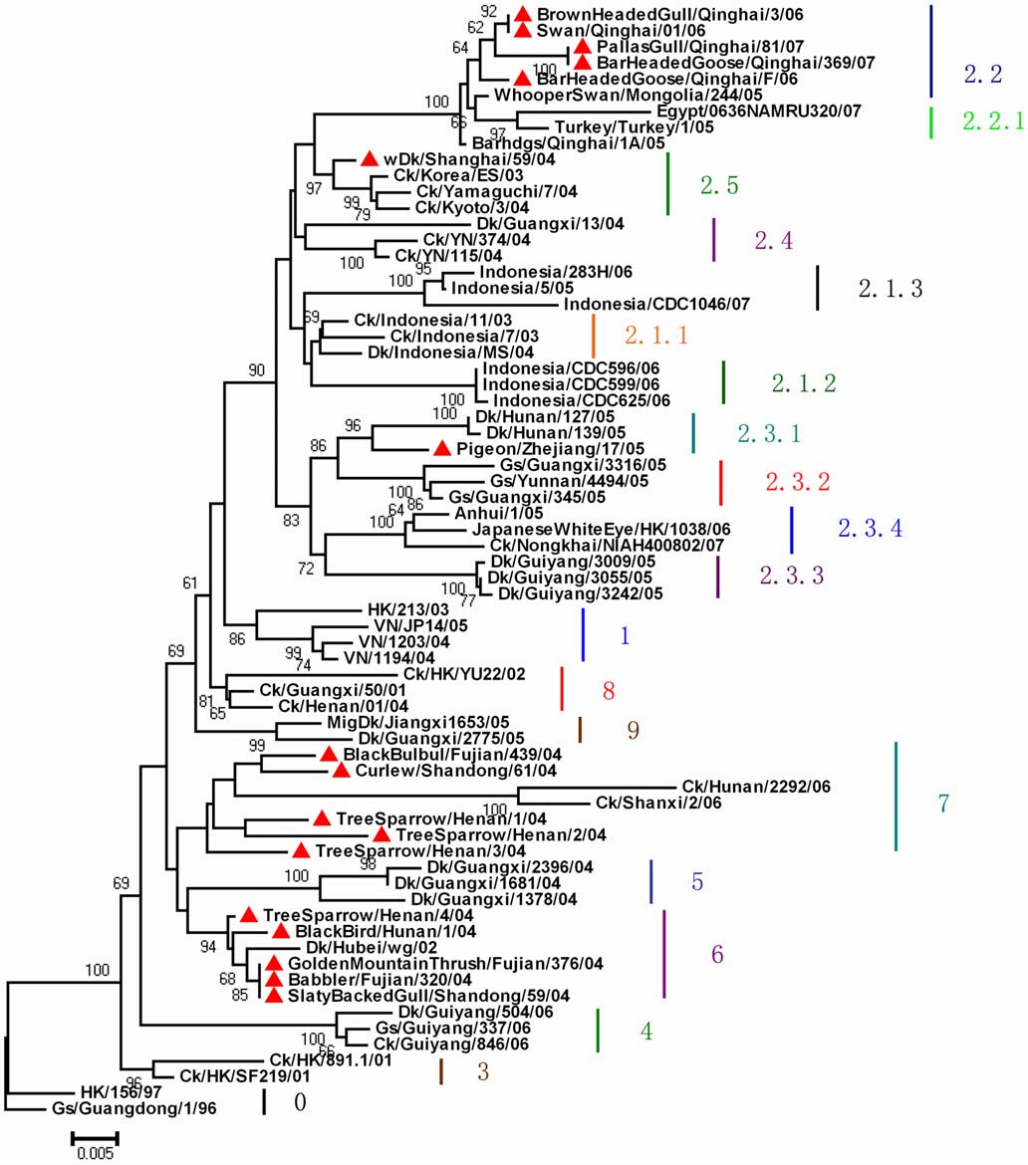
Phylogenetic tree of HA genes. The red solid triangles represent the 17 H5N1 viruses in wild birds. Neighbor-joining trees were generated using MEGA Version 4.0. Numbers below branches indicate bootstrap value percentages from 1,000 replicates (not shown if <60). Analysis was based on nucleotide 170 to 1012 (842 bp) of HA gene. The scale bar shows the mutation rate between each two sequences.

The phylogenetic tree of HA genes was constructed to determine the relationship with other H5N1 viruses (Figure 2). All the reference sequences were selected according to the latest denomination method by the World Health Organization [24]. As shown in Figure 2, the 17 viruses distributed in five clades (2.2, 2.3.1, 2.5, 6 and 7; 17 sequences have all been deposited in GenBank and the accession numbers are AY741213, AY741215, AY741217, AY741219, AY741221, DQ188905–DQ188908, DQ191688–DQ191689, DQ822563–DQ822565, FJ461725–FJ461726, FJ517645). Five virus isolated from Qinghai province were only distributed in the group of 2.2 and had short evolutionary distances with H5N1 viruses isolated in Qinghai in 2005, which suggested the HA genes in Qinghai were conserved.

## Discussion

The positive samples involved 24 species of six orders, among which the prevalence of H5N1 virus varied. The samples of Anseriformes had the highest prevalence and those of Passeriformes had the lowest prevalence (2.70% versus 0.36%, Pearson χ^2^-test, ρ<0.001). It was reported that H5N1 viruses were isolated from dead wild birds [13]–[17], which suggested that H5N1 virus could infect wild birds to cause death. Recently, it was also reported that some duck species suffered from severe disease upon experimental infection [25]. During the survey in China from 2004 to 2007, it was found that several species occasionally shown obvious sign of disease or death, from which the samples were H5N1 positive. Theses species included Bar-headed Goose (*Anser indicus*), Whooper Swan (*Cygnus cygnus*), Brown-headed Gull (*Larus brunnicephalus*), Pallas's Gull (*Larus ichthyaetus*), Tufted Duck (Aythya fuligula), Rock Dove (*Columba livia*), Tree Sparrow (*Passer montanus*). However, the number of theses species with signs of disease was very small and the mortality was closed to zero.

Moreover, a certain difference of prevalence was found between 14 provinces as shown in table 3. Qinghai had a higher prevalence than other 13 provinces combined (3.88% versus 0.43%, Pearson χ^2^-test, ρ<0.001) As shown in figure 1, most H5N1 positive provinces (Qinghai, Gansu, Shanghai, Zhejiang, Neimenggu, Shandong, Shannxi, Fujian, Hunan) had poultry or human H5N1 cases [26], [27], which suggested that these cases might be related with wild birds. Most of the samples in Qinghai province were collected from migratory birds (Table S1), which suggested that the high prevalence of Qinghai (3.88%) might be related to migratory birds. Qinghai province, located on the Qinghai-Tibet plateau of Northwest China, had special environmental ecology and various wildlife species. The reasons for the high prevalence in Qinghai province may be the special environmental ecology and the migratory bird habitat in Northwest China [13]–[16].

In the paper, we found the samples of 24 species were positive for H5N1 virus and the prevalence for Anseriformes was relatively high, which suggested that H5N1 virus might be maintained by wild birds, especially by water fowls. However, it was not excluded that H5N1 viruses were transmitted repeatedly from ongoing outbreaks in poultries to wild birds. In China, water fowl could be closely contact with poultries (duck, goose or quail) in the water environment. Excrements of poultries infected by H5N1 viruses could contaminate the living environment for water fowls. Moreover, the resident birds living around the farms could be infected by poultry H5N1 virus and carry virus from farm to the water environment around water fowls. H5N1 Viruses existed in the environment could infect water fowls, which could transmit H5N1 viruses when they are migrating. For example, Qinghai Lake is a major breeding site for migratory birds and did not have livestock breeding, but the range of migration for migratory birds included Xinjiang, Neimenggu, Gansu, Shannxi, Hunan, Xizang, Yunnan, where many poultry cases were reported from 2004 (Figure 1). During migrating process, water birds might be infected by poultries. Most birds had no signs of diseases or death after infected by H5N1 viruses. When wild birds came back Qinghai Lake, the habitat could be contaminated and continuous cross transmission between different species occurred.

Outbreaks of bird flu among migratory birds were reported in Qinghai province in 2005, 2006, 2009 [26]. However, no large-scale outbreak of bird flu was found among resident birds. In the paper, tree sparrows (*Passer montanus*) had a relative high prevalence (1.09%), which is similar to Pigeons (*Columba livia*; the prevalence is 1.05%). These resident birds often appeared in the cultivation zones of poultries and the habitats of water fowls and could come in contact with infected birds or infected environment. The analyzed results suggested that these resident birds had a high risk of transmitting of bird flu virus between wild birds and poultries [20]. Therefore, the chances of the contact between poultries and resident birds should be reduced as many as possible.

In the paper, the prevalence of H5N1 virus among wild birds in China were relatively higher than those in Europe or Africa [28]. There were three possible reasons for this difference: 1) The scale of livestock breeding for poultry in China is huge. The production organization mode is mainly rural household's scatter breeding, which increased the possibilities of contacts between wild birds and poultries. 2) Outbreaks in poultry were ongoing in China from 2003, which caused the repeated infection among wild birds. 3) Wild birds could carry H5N1 virus and contaminated habitats, which could cause continuous cross transmission between different species. The origin of H5N1 virus in wild birds was complicated and involved many factors (birds' live habit and ecology environment, H5N1 prevalence among poultries, the scale and organization model of livestock breeding, etc.).

As shown from the phylogenetic tree, the 17 strains isolated from wild birds distributed in 5 groups (2.3.1, 2.2, 2.5, 6 and 7), which suggested that genetic diversity existed among H5N1 viruses of wild birds and that the genotypes in wild birds in Chinese mainland was complicated [24], [29]. The five isolates from Qinghai were all within the group of 2.2 and it had a short evolutionary distance with the isolates obtained from Qinghai in the year 2005, which indicated the HA gene from Qinghai stains were conserved [15], [16]. It is reported that H5N1 viruses isolated from wild birds were in the clade 0, 1, 2.2, 2.3.1, 2.3.2, 2.3.4, 2.5, 3, 6, 7, 9 [17], [24]. Although most H5N1 viruses in wild birds could be regarded as occasional infection (single specie, single location, or single isolation time), clade 2.3.2 viruses were isolated from nonpasserine birds in Hong Kong, Japan, and Russia from 2004 to 2008 [17] and clade 2.2 viruses were isolated from wild birds in many countries in Asia, Europe, Africa from 2005 to 2007 [24], which suggested that H5N1 virus of clade 2.3.2 and clade 2.2 might be maintained by wild birds [17].

The outbreak of bird flu H5N1 subtype proved that our common knowledge on the flu in wild birds was still insufficient. It is very important to know if H5N1 virus existed in wild birds continuously and continuous monitoring in the field should be carried out.

## Materials and Methods

### Ethics Statement

All animals were handled in strict accordance with good animal practice as defined by Hubei Animal Welfare Council, China, and all animal work was approved by State Forestry Administration, China.

### Sample collection

We launched a sample collection about the wild birds living in the major lakes and wetlands covering 14 provinces in China from 2004 to 2007 (Figure 1). The samples were collected in five provinces in East China (Anhui, Fujian, Shanghai, Shandong and Zhejiang), three in Central China (Hubei, Hunan and Henan), three in Northwest China (Qinghai, Shaanxi and Gansu), two in Northeast China (Jilin, Heilongjiang), and one in North China (Neimenggu). All the samples were collected during the major migration period, lasting from March to April and from August to September each year.

From 2004 to 2007, we collected 14,472 wild bird samples in total, covering 56 wild bird species of 10 orders (Table 1). All the samples were cloacal swabs, organ tissues or fresh excrements collected from the gathering place of a bird flock of a single species. All the samples were put into small tubes containing transferring solution immediately, and then stored in liquid nitrogen container within 2 hours. The transport medium consisted of Hanks balanced salt solution/glycerol(10%), containing antibiotic (200 U/mL penicillin, 200 µg/mL streptomycin, 100 U/mL polymyxin B sulfate, 250 µg/mL gentamicin, and 50 U/mL nystatin).

### RAN extraction and identification of virus subtype

For cloacal swabs, RNA was extracted using QIAamp Viral RNA Mini kit (QIAGEN, Gmbh, Germany) according to the standard protocol. 140 µL transport medium were used to extract RNA, and then RNA was eluted by 50 ul elution buffer. For tissues and droppings, it was homogenized and centrifuged firstly. 140 ul supernatant liquor was used to extract RNA as above. RNA was stored in −80°C. Conventional reverse transcription polymerase chain reaction (RT-PCR) was performed using a one-step RNA PCR kit (Takara Bio, Dalian, China) to identify the subtype according to the standard protocol. The primer for reverse transcription of viral RNA genome was 5′-AGCAAAAGCAGG-3′. The primers for PCR was designed according to the WHO recommended manual for H5N1 identification based on conventional RT-PCR [30]: 5′- GCCATTCCACAACATMCACCC-3′ for forward primers 5′- CTCCCCTGCTCRTTGCTATG-3′ for reverse primers. For each run, the samples were prepared and processed in parallel with negative and positive controls. PCR products were routinely processed with 1% agarose gel electrophoresis. The expected length of the specific amplified fragment was about 0.2 kb.

### Isolation of virus

H5N1 positive samples were subsequently processed for virus isolation. Cloacal samples were diluted with phosphate-buffered saline (PBS) and centrifugation. Tissues and droppings were homogenized and centrifuged, and supernatant were collected. All positive samples were mixed with equal volume of PBS containing antibiotics (penicillin G, 40,000 IU/ml; streptomycin sulfate, 8000 IU/ml) overnight at 4°C, and then centrifuged and inoculated into the allantoic cavities of 10-day-old specific-pathogen-free embryonated eggs (Merial Ltd. Co.,Beijing, China). After incubation at 37°C for 24 to 72 h, the allantoic fluid of the inoculated eggs was harvested. All the experiments were performed in a Bio-safety level 3 laboratory.

### Sequence analysis

According to the PCR protocol described previously [31], hemagglutinin genes were further sequenced to determine the relationship with other H5N1 strains by using the Amersham ET Dye Terminator kit (Amersham Pharmacia Biotech, Piscataway, NJ) and the ABI PRISM 370 DNA sequencer (PE Applied Biosystems, Foster City, CA). All sequence data was edited by BioEdit 7.0.1 and aligned by Clustal X 1.8. Neighbor-joining trees were generated using MEGA 4.0. All the reference sequences were selected according to the latest denomination method by the World Health Organization [24].

### Statistics

The Pearson χ^2^-test were used for analysis of the dataset used in this study.

## Supporting Information

Table S1 Bird species involved in the study. Table S1 includes data on all species in which H5N1 virus was detected by RT-PCR, including geographical sampling location and sample size.
(0.07 MB XLS)
